# Sire risk factors for vertical transmission of *Leishmania infantum* by the dam

**DOI:** 10.1371/journal.pntd.0014303

**Published:** 2026-05-04

**Authors:** Kayla R. Duxbury, Luciana M. Richer, Christine A. Petersen

**Affiliations:** 1 Department of Epidemiology, College of Public Health, University of Iowa, Iowa City, Iowa, United States of America; 2 Department of Veterinary Biosciences, College of Veterinary Medicine, The Ohio State University, Columbus, Ohio, United States of America; Centro de Pesquisa Gonçalo Moniz-FIOCRUZ/BA, BRAZIL

## Abstract

*Leishmania (L.) infantum* causes visceral leishmaniasis (VL) in humans across the Mediterranean basin and in Central and South America. VL is a zoonotic disease, with dogs as the predominant domestic reservoir. Traditionally, this disease is transmitted via sand flies of the genus *Phlebotomus* or *Lutzomyia* as vectors. Case reports of transmission in non-endemic areas have increased, where transmission is predominantly vertical, sexual, or bloodborne. *L. infantum* has been shown to be enzootic in hunting hounds within the United States (U.S.), with no indication of vector borne transmission. In this population, there is very high risk of parasite spread if the dam’s diagnostic status is positive. In comparison, very little is known about the risk if the sire not the dam is positive for *L. infantum*. This is a retrospective cohort study of 24 U.S. hunting hound sires’ *L. infantum* exposure between 2013 and 2022 and the resultant evidence of infection in their 183 pups over that time. Offspring from sires who tested serologically positive for *L. infantum* during the year of birth had 1.59 times the risk of becoming diagnostically positive via serology or molecular detection for *L. infantum* during their lifetime (RR: 1.59 95% CI: 1.15-2.20 p-value: 0.0046) when compared to outcomes in pups from diagnostically negative sires. The basic reproductive number (R_0_) for the risk of the sire to indirectly transmit *L. infantum* to the pup within this cohort was 3.71. These results demonstrate the impact of sire’s infection on transmission of *L. infantum* to offspring. There is a need for control efforts that address non-vectorial transmission from both parents. Control efforts targeting vector borne transmission of canine leishmaniosis in endemic countries are also needed.

## Author summary

*Leishmania (L.) infantum* is a parasite that infects both humans and animals, causing Leishmaniasis in humans and Canine leishmaniosis (CanL) in dogs. This disease is deadly and is commonly spread by sand flies between dogs, also infecting humans or other animals. Case reports in areas where this parasite is not common have reported parasite spread from dog-to-dog through sex and fighting, as well as blood transfusions and from dam to offspring. *L. infantum* has been found in hunting dogs across the United States, without evidence of sand fly infection. Previous research has investigated how the dam’s diagnostic status impacts her offspring’s risk of becoming diagnostically positive for *L. infantum*, but no studies have identified risks associated with a positive sire. Samples were previously collected and sent in for diagnostic testing between 2007 and 2022. Data from a total of 183 pups born to 24 sires from these collections were analyzed. Offspring from sires who tested serologically positive for *L. infantum* during the year of birth had 1.59 times the risk of becoming diagnostically positive for *L. infantum* during their lifetime (RR: 1.59 95% CI: 1.15-2.20 p-value: 0.0046). The basic reproductive number (R_0_) for the risk of the sire to transmit *L. infantum* to dams within this cohort was 3.71. These results demonstrate the capacity of the sire to indirectly transmit *L. infantum* to offspring. It is important to consider how breeding animals known to be infected with parasites impacts spread to future generations and continuation of infectious animals for onward spread to other dogs and people. Taking precautions to prevent breeding infected animals prevents future spread of *L. infantum*.

## Introduction

*Leishmania (L.) infantum*, is found globally, but predominately found in parts of South America, the Mediterranean basin, and West and Central Asia [1]. Female sand flies from the species *Phlebotomus ariasi*, *Phelbotomus perniciosus*, and *Lutzomyia longipalpis* are the main vectors for transmission of *L. infantum* from a reservoir host, predominantly dogs, to a new host [2]. Infection with *L. infantum* leads to visceral leishmaniasis (VL) in humans and canine leishmaniosis (CanL) in dogs [1–3]. VL can be fatal, often resulting in death within two years of infection [3,4]. To control zoonotic VL, national health policies from various countries have recommended culling any *L. infantum* seropositive dog, though no evidence to shows that culling of seropositive dogs has an impact on the number of human cases of zoonotic VL [5–7]. Opting to control canine *L. infantum* infection by preventing sand fly bites via prophylactic insecticides and bed nets to prevent transmission has become the more approved method to in turn prevent more zoonotic VL cases within the human population [2,8].

Dogs are the major zoonotic reservoir for *L. infantum* globally- [2,9]. Due to animal transportation for trade, travel, and rehoming, dogs are also the main source of *L. infantum* in non-endemic countries [6,9,10]. Dogs who spend more time outside, such as hunting, guarding, and wandering dogs have been shown to have higher odds of being infected with *L. infantum* [11]. *L. infantum* is spread between dogs in endemic areas through the bite of infected female sand flies, but horizontal transmission - through dog bites, blood transfusions, organ transfusions and also vertical and venereal transmission are seen [10]. Roughly 10% of dogs who are infected will go on to develop CanL, while another large portion presents as subclinical and able to spread the infection [10].

CanL was first found in the United States in 1980 in a dog with no travel history outside of the United States [12,13]. By 1999, outbreaks of CanL were found among US hunting dogs [13]. Since then, studies have looked at the prevalence of *Leishmania* within this population. Due to educational pamphlets and new studies being conducted, the prevalence of *Leishmania* infections decreased between 2007 and 2015, with some fluctuations [14]. As of 2015, an active testing cohort saw 8.9% testing positive, while a passive testing cohort saw 2.2% dogs testing positive [14]. Genetic markers of *L. infantum* within U.S. hounds compared to variations from areas around the world found U.S. parasites varied from those originating elsewhere, pointing to clonal reproduction, rather than sexual via meiosis in the sand fly [13]. Transmission within this dog population was instead vertical, transmitting from dam to pup [13]. A study conducted between 2010 and 2013 concluded that *L. infantum* circulating within the hound population was still able to develop within *L. longipalpis* and infect healthy hamsters, indicating potential for a vector-transmission within the U.S. [15].

Multiple studies have shown evidence of canine maternal vertical transmission [16–20]. One study found that offspring of a dam who tested positive for *L. infantum* via serology or qPCR have 13.84 times the risk of becoming diagnostically positive for *L. infantum* during its lifetime [20].

Far fewer studies have investigated the role of the father as a risk factor for indirect transmission of *L. infantum* to their offspring. *Leishmania* spp. parasites have been found within seminal fluid of serologically positive dogs [18,21,22]. A case report of a 6-year-old Cane Corso from Italy living in Northern France in good condition with an unremarkable examination who had reports of infertility after multiple successful breeding attempts was found to have chronic prostatitis and confirmed *L. infantum* or *L. donovani* within the seminal plasma [22]. *Leishmania* spp. is not always shed within semen, as in the Silva et al., only 13 of the 15 serologically positive dogs had evidence of *Leishmania* kDNA in semen and even then, only 2 dogs had all 3 semen samples test positive, while the other 11 dogs had random and intermittent positivity [18,23].

Understanding the role of the sire in transmission to pups, thus resulting in vertical transmission, has implications for canine health. Knowing how the sire’s diagnostic status impacts that of the offspring can spur changes in current breeding practices to reduce the number of infected dogs.

## Materials and methods

### Ethics statement

As this was a retrospective study, there was no live animal work for this study. All previous animal use involved in this work was approved by the University of Iowa and in some rare older cohort instances, Iowa State University, Institutional Animal Care and Use Committee and was performed under the supervision of licensed and, where appropriate, board-certified veterinarians according to International AAALAC accreditation standards.

### Study design

A retrospective cohort study based on data collected regarding *L. infantum* infection and exposure within U.S. hunting dogs since the 1999 outbreak was completed [24,25]. Two sets of sires, those that were and those that were not ELISA or qPCR positive for *Leishmania* were identified to assess sire exposure. *L. infantum* diagnostic testing from all offspring of these two respective groups were identified and assessed to determine their *Leishmania* diagnostic status. All historical data were collected from studies conducted by Center for Disease Control and Prevention [24,25], and the Petersen laboratory at Iowa State University and University of Iowa [14,15,20,26–29]. This study compiles data collected from 2004 to 2022.

### Animals

Yearly active surveillance of the U.S. Foxhound, Beagle, and Basset hound hunting kennels was conducted from 2004 to 2022. During routine visits, licensed veterinarians collected 1–5 mL of whole blood and serum from all dogs older than 9 months. For passive surveillance, samples from dogs 9 months of age or older were submitted to our laboratory from hunting kennels for routine *Leishmania* diagnostic testing. All assays were performed within one month of sample collection or shipping. Demographic information regarding year of birth, sex, and age were recorded. All hunting kennels bred their animals naturally.

### Real time quantitative polymerase chain reaction

DNA was isolated from canine whole blood samples collected in heparinized or ethylenediaminetetraacetic acid (EDTA) via the QIAmp DNA Blood Mini Kit (Qiagen, Valencia, CA) per manufacturer protocol. The quality and quantity of DNA was assessed using NanoDrop 2000 (Thermo Scientific, Waltham, MA).. All qPCR assays included blood spiked with 10^6^ *L. infantum* parasites to act as positive controls, and non-hound healthy dog samples for negative controls. Samples from 2004 to 2006 were tested at Center for Disease Control and Prevention via *Leishmania* specific IFAT as previously published [24,25]. From 2007 to 2011, kinetoplastid primers (kDNA) and probe targets were used. The primers and probe sequences were: kDNA Forward 5’-CCGCCCGCCTCAAGAC, kDNA Reverse 5’-TGCTGAATATTGGTGGT TTTGG (Integrated DNA Technologies, Coralville, IA), kDNA probe, 5’-6FAM-AGCCGCGAGGACC-MGBNFQ (Applied Biosystems, Foster City, CA). Based on findings that these sequences can wobble over time [31], we then moved to a more stable sequence. From 2012 to 2022, ribosomal primer (18S rRNA) and probe targets were utilized. The sequences were as follows: 18S rRNA Forward 5’-AAGTGCTTTCCCATCGCAACT, 18S rRNA Reverse 5’-CGCACTAAACCCCTCCAA (Invitrogen, Life Technologies, Grand Island, NY), 18S rRNA probe: 5’ 6FAM-CGGTTCGGTGTGTGGCGCC-MGBNFQ (Applied Biosystems, Life Technologies, Grand Island, NY). In 2022, the reverse ribosomal primer was updated to 5’ -GACGCACTAAACCCCTCCAA (Invitrogen, Life Technologies, Grand Island, NY). The positive qPCR results were based on amplification cycles (Ct) above the basal threshold level [14,20,26,27,30]. Assays were performed on ABI 7000 systems until 2016 and forward, ABI 7900 systems (Applied Biosystems). Analysis was performed using ABI 7000 System SDS Software and ABI 7900 HT Sequence Detection Systems Version 2.4.1. (Applied Biosystems). Starting in 2019, assays were performed on QuantStudio 3 with analysis being conducted using Design & Analysis 2 Software (ThermoFisher, Waltham, MA).

### Serology testing

Serological status was determined by immunofluorescent antibody test (IFAT) or the Dual Path Platform Canine Visceral Leishmaniasis (DPP CVL) assay (Chembio Diagnostic Systems Inc., Medford, NY) or Soluble *Leishmania* Antigen enzyme-linked immunosorbent assay (ELISA) in different periods of the study. From 2007 to 2014, IFAT was performed by the Division of Parasitic Diseases at the Centers for Disease Control and Prevention (CDC) as previously described [24,32]. CDC samples reported positive for serum dilutions equal to or above 1:64 in 50% *L. infantum* promastigotes. These tests were performed blindly and were repeated four times at each dilution. From 2015 to 2017, the DPP CVL assay was utilized. This rapid point-of-care assay detected *Leishmania*-specific antibodies in dog serum. All positive or questionable samples were confirmed using the Chembio microreader system. After 2017 until 2022, Soluble *Leishmania* Antigen (SLA) ELISA was implemented. SLA antigen is soluble antigens isolated after freeze – thaw to lyse *L. infantum.* Using a plate reader, OD450 absorbance was used to determine outcome. Samples were considered positives if the readings are above the cut-off (average absorbance of negative controls plus 3 standard deviations) [33–35].

### Statistical methods

**Univariate analyses** were performed to determine the unadjusted relative risk values for the proportion of male puppies and the sire’s diagnostic status during the year of birth. Mann-Whitney U test was used to compare sire’s age and litter size between positive and negative groups as age and litter size are not normally distributed. Fisher’s exact test was used to compare categorical variables between diagnostic groups. For feasibility reasons, the sire’s diagnostic status for qPCR and serology were determined as status the year of pup’s birth, as the exact date of insemination is not known.

**Multivariable logistic regressions** were performed to determine the adjusted relative risk of a pup testing positive for *L. infantum* during its lifetime via either serology or qPCR. The sire’s diagnostic status was assessed in different ways within three models. The first model included independent variables for the overall diagnostic status of the sire (never diagnostically positive compared to ever diagnostically positive), the sire’s age at time of birth (younger or equal to 7 years of age compared to older than 7 years of age), and the sex of the puppy (female compared to male). To further assess the impact of the sire’s diagnostic status, a second model was run that included the sire’s diagnostic status the year the pups were born along with sire’s age and pup’s sex. A third model was created using the sire’s qPCR and serology status as two different explanatory variables during the year of birth in addition to the sire’s age and pup’s sex. A p-value less than 0.05 was determined to be statistically significant. Each model was fit assuming a binomial distribution with a log-link function as there was expected to be a small risk associated with the outcome and odds ratio would overestimate the association between the exposure and outcome, relative risk was determined to be the better option. Calculating risk and risk ratios was more beneficial for the community that would use this information. Kaplan-Meier time to event analysis was performed to assess the degree to which the sire’s diagnostic status altered time to pup’s diagnostic status within three models (ever positive, qPCR positive, and seropositive).

**Basic reproductive number** was calculated using sires who were ever diagnostically positive. Foxhounds are a large breed dog, who have average litter sizes of 7 [36]. Using the average litter size, the number of puppies in each litter that would become diagnostically positive for *L. infantum* was determined as the basic reproductive number of vertical transmission in foxhounds. The equation R_0_ = average litter size x proportion of infected pups was used. This is a deterministic individual-level model and assumes littermates do not affect outcome of each individual offspring.

For all analyses, as previously described, *L. infantum* diagnostic status was determined for each sire as “ever diagnostically positive” or “never diagnostically positive” for *Leishmania*. A positive status was determined as qPCR and/or seropositive at any point of the sire’s lifetime [20].

**Loss to follow up and exclusion criteria.** This cohort consists of one hundred eighty-four pups, twenty-four sires, and twenty-six dams. Eight pups were excluded from analyses after litter demographic assessments were conducted due to incomplete serology or qPCR results. Ever and never diagnostic statuses for pups, sires, and dams were positive if the dog had one test indicating positive. This required testing results for the dam, sire and pups prior to the pups’ birth (within the prior year). For a dog to be considered never positive, both serology and qPCR results had to be negative. Two pups were missing any serological status. Four sires were missing qPCR diagnostic status prior to pups’ birth (37 pups). Four sires were missing serological diagnostic status prior to pups’ birth (41 pups). Five dams were missing qPCR diagnostic status prior to pups’ birth (34 pups). Eight dams were missing serological diagnostic status prior to pups’ birth (56 pups). Four dams were missing qPCR diagnostic status before and after pups’ birth (20 pups). Two dams were missing criteria to give a never positive diagnostic status (10 pups). When critical data were missing these dogs were removed from the dataset. All breeder pair information was provided by the kennels.

Using an alpha error probability of 0.05, power of 0.80 and total sample size of 183, an effect size of 0.2071 was calculated for this study. We analyzed data from an existing sample and computed the minimal detectable effect size based on the sample size using 80% power. The minimal detectable effect size was calculated using Cohen’s w with G*Power 3.1.9.7. [37].

All statistical analyses were performed using SAS 9.4 (SAS Institute, Cary, NC, USA).

## Results

### Study cohort demographics based on Sire’s diagnostic status

To assess the impact of sire’s status on risk of vertical transmission of *L. infantum*, a retrospective cohort study examined test results from 183 dogs born to 24 sires. Eight offspring were removed from the analysis after sire demographic data analyses were conducted due to missing complete diagnostic statuses. Sixteen of the sires (16/24 – 66.7%) were found to test positive for *Leishmania* at some point during the study, while eight sires were diagnostically negative for *L. infantum* throughout their lifetime. There were multiple instances of polygyny, mating of a male with more than one female, and for one dog, polygyny occurred within one breeding season. Both *Leishmania* positive and negative sire groups had slightly over 50% male puppies (55.22% vs. 59.18%) (Table 1).

**Table 1:** Demographics based on Sire’s *Leishmania* diagnostic status. Sire’s ever positive diagnostic status was determined as sire ever positive via qPCR or serology.

Variables	Sire ever *L. infantum* dx Positive (n = 16)	Sire L. *Infantum* dx Negative (n = 8)
Average age of sire during year of birth ±SD (Min-Max)	4.19 ± 2.71 (2-10)	5.11 ± 1.36 (4-8)
Proportion of male puppies Proportion %, N	55.22, 74	59.18, 29
Average litter size ±SD (Min-Max)	7.24 ± 2.9 (1-15)	5.78 ± 2.22 (2-8)
Proportion of puppies *L. infantum* diagnostic positive ever* Proportion %, N	53.08, 69	51.11, 23

*Four pups missing diagnostic data from each group (8 total).

There was no significant difference in demography between pups *L. infantum* infected or non-infected sires regarding sire’s age during the year of pups birth and litter size based on univariate comparisons. Further analysis of unadjusted relative risks showed no statistically significant risk for male offspring compared to female offspring and sire’s diagnostic status both during the year of birth via qPCR and serology and throughout the sire’s lifetime. The qPCR and serology statuses each independently had a larger impact on the unadjusted risk compared to the combined sire’s ever diagnostic positive status (Table 2).

**Table 2:** Univariate analysis of risk factors for impact of sire’s infection on transmission to Pup. Pup ever diagnostically positive is the outcome with sire’s ever positive diagnostic status as the exposure. Sire’s ever positive diagnostic status was determined as sire ever positive via qPCR or serology during the year of birth. Number of pups with missing sire diagnostics: 111, 132, 263, 284, 375, and 416. + Diagnostic status during year of birth.

Variables	Pup ever *L. infantum* dx Positive (n = 92)	Pup never *L. infantum* dx Positive (n = 83)	P-value	Unadjusted Relative Risk
Average age of sire year of birth ±SD (Min-Max)	4.57 ± 2.27 (2-10)	4.36 ± 2.49 (2-10)	0.2992	N/A
Proportion of male puppies Proportion %, N	55.43, 51	55.42, 46	0.1210	1.00
Average litter size ±SD (Min-Max)	8.24 ± 3.40 (1-15)	7.58 ± 2.08 (3-15)	0.7016	N/A
Proportion sire *L. infantum* qPCR positive^+^ Proportion %, N	29.63, 24^1^	38.60, 22^3^	0.0796^5^	0.84
Proportion sire *L. infantum* seropositive^+^ Proportion %, N	51.90, 41^2^	41.82, 23^4^	0.0728^6^	1.18
Proportion sire ever *L. infantum* dx positive^+^ Proportion %, N	75.00, 69	73.49, 61	0.1338	1.04

### Multivariable analysis models

A series of three binomial logistical regression models were created to determine if there are any sire related risk factors associated with vertical transmission of *Leishmania* to pups. We had previously found that the risk of death in an infected hound from all causes became significant after 6 years of age [30], and it is well established that there is an increased biological risk of infection in males [38,39]. The first model, A, used explanatory variables of overall diagnostic status of the sire (never diagnostically positive compared to ever diagnostically positive), the sire’s age during the year of birth (younger or equal to seven years of age compared to older than seven years of age), and the sex of the puppy (female compared to male). The second model, B, used explanatory variables of the sire’s diagnostic status at the year of pup’s birth, the sire’s age during the year of birth, and sex of the puppy. The final model, C, used explanatory variables of the sire’s qPCR and serology statuses and sire’s age during year of birth and sex of puppy. Via model B, we found that pups born to a sire that was serologically positive for *Leishmania* during the year of the pup’s birth had 1.57 times the risk of becoming positive for *Leishmania* throughout their lifetime compared to pups from negative sires (Adjusted RR: 1.57, 95% CI: 1.14-2.16, p-value: 0.0055, Table 3).

**Table 3:** Binomial regression with log-link (RR) analysis for risk factors associated with impact of sire’s on *Leishmania* venereal transmission. Model A: Explanatory variables include overall diagnostic status of the sire (never diagnostically positive compared to ever diagnostically positive), the sire’s age during the year of birth (younger or equal to 7 years of age compared to older than 7 years of age), and the sex of the puppy (female compared to male). Model B: Explanatory variables are sire’s diagnostic status at the year of pup’s birth, the sire’s age during the year of birth, and sex of the puppy. Model C: Explanatory variables include sire’s qPCR and serology statuses and age during year of birth and sex of puppy. ^1^40 observations missing due to missing either serologic or molecular sire diagnostic status year of birth. ^2^47 observations missing due to missing either serologic or molecular sire diagnostic status.

Model	Variables	Sample Size N = number of sires n = number of puppies	Binomial Regression with long-link (RR)	95% CI	p-value
A	Sire ever *L. infantum* dx positive	N = 24 n = 175	1.04	(0.75, 1.44)	0.8138
B	Sire dx positive during year of birth	N = 20 n = 135^1^	1.34	(0.99, 1.82)	0.0612
C	Sire qPCR positive year of birth	N = 19 n = 128^2^	0.76	(0.55, 1.06)	0.1090
C	Sire seropositive year of birth	N = 19 n = 128^2^	1.57	(1.14, 2.16)	0.0055

The sire’s qPCR status during the year of birth had a 95% confidence interval between 0.55 and 1.06 and the sire’s diagnostic status during the year of birth having a 95% confidence interval between 0.99 and 1.82 indicates that the variable is scientifically interesting to better understand the role of the sire’s diagnostic status for future research with larger sample sizes (Table 3).

### Stratification

When the dam’s diagnostic status during pregnancy was used in the multivariable analysis model as stratification, pups born from seropositive parents are at a higher risk of testing positive for *L. infantum* during their lifetime (Tables 4 and 5). An additional 10 pups were removed due to missing diagnostic status of the dam with 67 pups having a never diagnostic positive dam and 98 pups having an ever positive dam. Pups born to a sire that serologically positive for *Leishmania* during the year of the pup’s birth and a dam that is ever positive had 1.58 times the risk of becoming positive for *Leishmania* throughout their lifetime (Adjusted RR: 1.58, 95% CI: 1.04-2.40, p-value: 0.0333, Table 5).

**Table 4:** Multivariable Logistic Regression Analysis for Sire Risk Factors Associated with *Leishmania* Venereal Transmission with dam negative. Model C: Explanatory variables include sire’s qPCR and serology statuses and age during year of birth and sex of puppy. ^1^ 28 missing observations due to missing either serologic or molecular sire diagnostic status.

Model	Variables	Sample Size N = number of sires n = number of puppies	Adjusted RR of pup lifetime exposure	95% CI	p-value
C	Sire qPCR positive year of birth	N = 9 n = 39^1^	0.19	(0.02, 1.52)	0.1183
C	Sire seropositive year of birth	N = 9 n = 39^1^	3.40	(0.53, 21.64)	0.1950

**Table 5:** Multivariable Logistic Regression Analysis for Risk Factors Associated with sire’s impact on *Leishmania* Venereal Transmission with dam positive. Model C: Explanatory variables include sire’s qPCR and serology statuses and age during year of birth and sex of puppy. ^1^16 observations missing due to missing either serologic or molecular sire diagnostic status.

Model	Variables	Sample Size N = number of sires n = number of puppies	Adjusted RR of pup lifetime exposure	95% CI	p-value
C	Sire qPCR positive year of birth	N = 16 n = 82^1^	1.52	(0.89, 2.61)	0.1238
C	Sire seropositive year of birth	N = 16 n = 82^1^	1.58	(1.04, 2.40)	0.0333

### Risk of pup positivity over time

**Fig 1:**
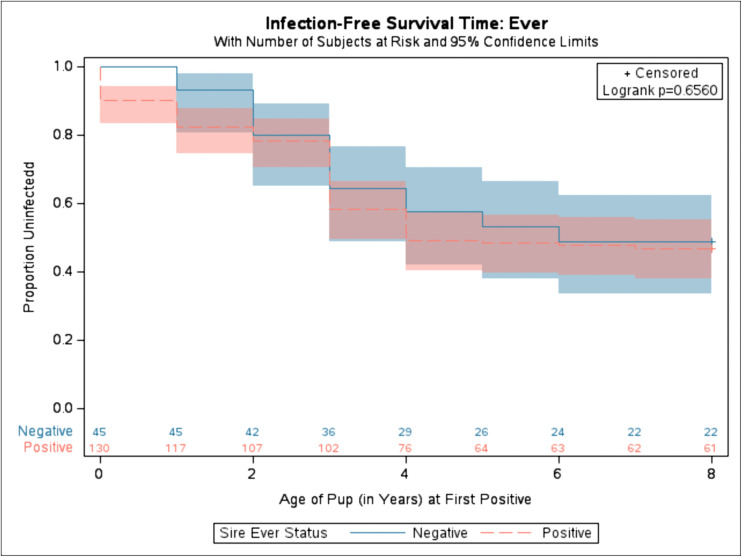
Kaplan-Meier time to offspring diagnostic positive based on Sire’s Ever diagnostic status. Proportion of uninfected refers to the proportion of dogs that were diagnostically negative via real-time PCR and serology. Solid blue represents the diagnostic status of pups from sire who were diagnostically negative via both qPCR and serology during their lifetime. Dashed red represents the diagnostic status of pups from sire who were diagnostically positive at any point during their lifetime via qPCR or serology. Shaded area represents variance around the mean. Number at risk displayed along x-axis. Log-rank test was used. (Chi-Square: 0.1984, p-value: 0.6560).

To better assess when dogs became diagnostically positive for *Leishmania*, Kaplan-Meier time to event analyses were conducted. To visualize the infection-free survival time, the age at which a pup first became positive via qPCR or serology was used. This was compared between the groups of sire ever versus never positive for *Leishmania*. It was not statistically significant for dogs born to positive (red dashed line) sires to have different ages of first diagnostic positives compared to dogs born to negative (blue solid line) sires (**Fig 1**).

**Fig 2:**
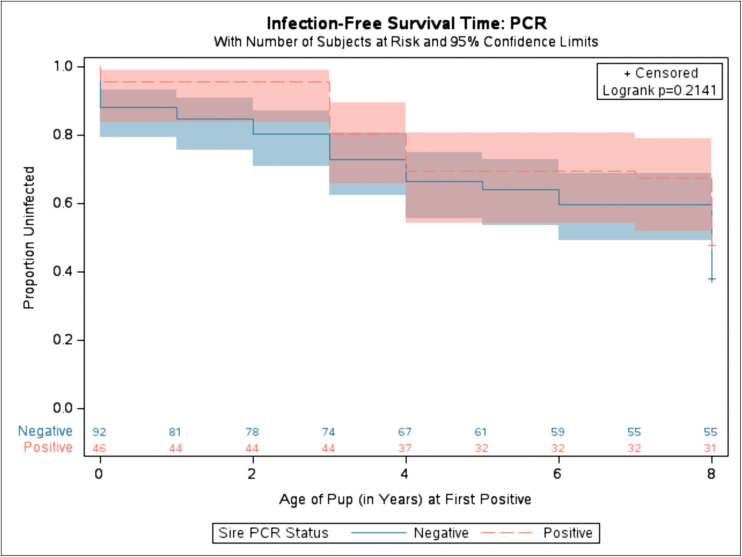
Kaplan-Meier time to offspring diagnostic positive based on Sire’s qPCR diagnostic status during the year of birth. Proportion of uninfected refers to the proportion of dogs that were diagnostically negative via qPCR. Solid blue represents the diagnostic status of pups from sires who were diagnostically negative before birth. Dashed red represents the diagnostic status of pups from sires who were diagnostically positive via qPCR before birth. Shaded area represents variance around the mean. 37 observations were excluded due to missing sire’s qPCR diagnostic status before birth. Number at risk displayed along x-axis. Log-rank test was used. (Chi-Square: 1.5437, p-value: 0.2141.

We further analyzed the infection-free survival times for qPCR status of the sire during the year of birth. The sire’s status (positive vs. negative) was utilized. As the findings were not significant, dogs born to positive (red dashed line) sires have ages of first qPCR positive that are not significantly different from dogs born to negative (blue solid line) sires (**Fig 2**).

**Fig 3:**
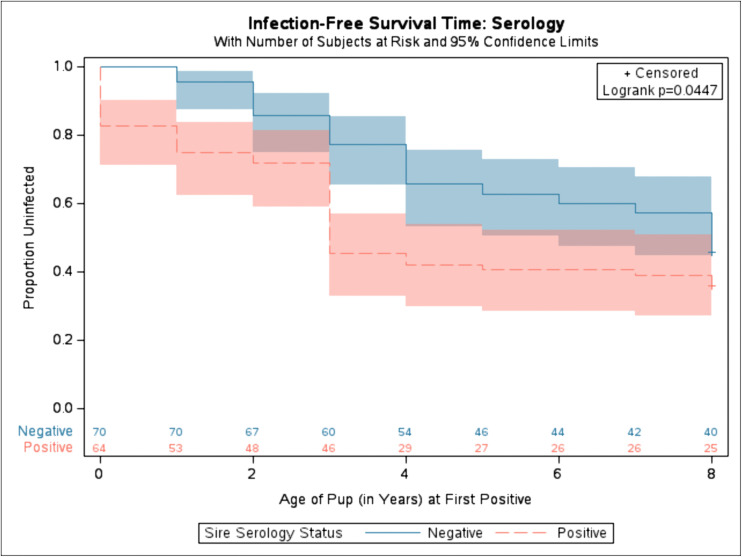
Kaplan-Meier time to offspring diagnostic positive based on Sire’s serology status during the year of birth. Proportion of uninfected refers to the proportion of dogs that were diagnostically negative via serology. Solid blue represents the diagnostic status of pups from sires who were diagnostically negative before birth. Dashed red represents the diagnostic status of pups from sires who were diagnostically positive via serology before birth. Shaded area represents variance around the mean. 41 observations were excluded due to missing sire’s serological status before birth. Number at risk displayed along x-axis. Log-rank test was used. (Chi-Square: 4.0301, p-value: 0.0447).

Based on the previous finding that sire serologic status during the year of birth was a significant variable in the pup becoming diagnostically positive during its lifetime, sire’s serological status (positive vs. negative) was utilized. The offspring born to sires who were seropositive (red dashed line) during the pup’s year of birth were significantly more likely to become seropositive for *Leishmania* at younger ages than offspring from sires that were negative (solid blue) (Fig 3, chi-square 4.0301, p-value 0.0447).

### Basic reproductive number

The basic reproductive number for venereal transmission of *Leishmania* is important to better provide rationale to institute control measures towards breeding. R_0_ was calculated based using this study population. An average of 53.08% of pups born to a sire ever diagnostically positive for *Leishmania* became positive during their lifetime (Table 1). Using an average litter size of our population, ~ 7, we calculated the average R_0_ to be 3.71. For this cohort, the R_0_ value is also able to be further considered in conjunction with the dam’s diagnostic status. A total of 32.65% of pups born to a sire ever diagnostically positive and a never positive dam for *Leishmania* became positive during their lifetime, giving an average R_0_ for this group to be 2.29. An average of 66.20% of pups born to a sire and dam both ever diagnostically positive for *Leishmania* became positive during their lifetime resulted in an average R_0_ for this group to be 4.62.

## Discussion

A retrospective cohort study was performed to assess potential risk factors associated with venereal transmission of *L. infantum* and to establish a basic reproductive number for the population. Through our analyses, the sire’s serologic diagnostic status during the year of pup’s birth for *L. infantum* was found to be a statistically significant risk factor for the offspring to become *Leishmania* positive during its lifetime. When our data is further stratified by dam’s diagnostic status, a positive dam and serologically positive sire during the year of birth are a statistically significant risk factor for the offspring to become *Leishmania* positive during its lifetime.

Using both ELISA and qPCR allowed us to gather a better understanding of current infection status of the dogs as well as immune response. Our qPCR was reliant on current infection. With dogs that have a low parasite load, there is the potential not to see a result indicating active infection but is sufficient to activate an immune response and be seen via ELISA.

Paternal venereal transmission for *L. infantum* is a widely understudied area of research. Although through our interaction with breeding kennels of hunting hounds we have heard many times they believed that the sire not dam was likely the source of infection to a litter, we had not looked at this specifically prior to this study. To consider how the sire could transmit *L. infantum* to his offspring, we considered that a localized infection within the uterus of a dam could result from breeding with an infected sire that had parasite-containing semen. If the infection is only localized to the uterus and is not systemic, it may have been diagnostically undetectable in the dam but passed from the dam to pup. Future research into breeding pairs and sexual transmission from male to female within the dog population is important better understand how to prevent future spread of *L. infantum* during breeding.

This study has potential limitations. The primary limitation for this study is that a large number of pups were lost to follow up. This study was from data gathered from yearly tested dogs within a handful of hunting kennels. To keep diversity within the larger hunting dog population, dogs are drafted and traded between kennels, leading to difficulty tracking a dog’s infection status throughout their entire lifetime. A second limitation is that horizontal transmission cannot be ruled out as a potential route of transmission within the kennels due to communal housing with dogs with the potential to bite and have blood to blood exposure, leading to bloodborne transmission of *Leishmania*. A third limitation is the occurrence of false-positives and false-negatives result due to the timing of blood collection, as parasite load in the blood stream is transient [40]. Traditional confirmation of the disease through tissue biopsy is not possible in these dogs because of the associated financial costs and the risk related to anesthesia.

Within this study, there were multiple cases of offspring testing positive, while both sire and dam tested negative throughout their lifetimes. In two of the cases, a *Leishmania* seropositive pup was born to 2 separate sets of parents who were all diagnostically negative for *Leishmania* via qPCR and serology throughout their lifetime which could indicate potential horizontal transmission. The third instance of this occurred with a litter of 7 pups born to a sire and dam both diagnostically negative via qPCR and serology throughout their lifetime, 4 pups tested positive for *Leishmania* via qPCR and serology, with 1 additional pup testing positive via qPCR only. This potentially indicates a localized infection in either the sire or dam that was spread to the offspring through venereal transmission or through horizontal transmission.

Two sires within this study had over half of their offspring testing positive. The first sire tested negative via qPCR before his first litter. No serology result was available for this litter. He went on to sire 2 more litters and had positive qPCR and serology results before birth for each. For his first litter, he was bred with a dam of unknown diagnostic status. This litter had 3 out of 4 pups testing positive within their lifetime, with one additional unknown pup – a pup that was reportedly born but transferred to another kennel before we did any annual testing. For his second litter, he was bred with a dam who was positive via qPCR with an unknown serology. 6 out of 7 tested pups from this litter were positive for *Leishmania* with 2 additional pups having an unknown status. In his final litter, he was bred with a dam negative for *Leishmania* via qPCR and an unknown serology. This litter had 4 pups testing positive for *Leishmania* during their lifetime, with the remaining 3 pups remaining negative. This sire had 13 pups out of a total of 21 pups become diagnostically positive within their lifetime. The second sire within the study was bred with 2 different dams within the same year. The sire was negative via qPCR, but positive on serology for *Leishmania*. Both dams tested negative via both qPCR and serology for *Leishmania*. The first litter had 4 out of 6 total dogs testing positive throughout their lifetime. The second litter had 2 out of 6 total dogs testing positive.

The basic reproductive numbers calculated are specific to hunting hounds and may have limited generalizability because they were calculated using an individual level model, calculating the number of pups within one litter that a sire infected. A previous study looking at the vertical transmission calculated an R_0_ for pups from dams who are diagnostically positive to be 4.16 [20]. With basic reproductive numbers of 3 for the entire cohort and 2 for pups born to mothers who never tested positive for *L. infantum* throughout her lifetime (rounded to whole numbers to indicate number of puppies within a litter), there is indication that the parasite can persist within the population just based on vertical transmission [41]. The 1.43-fold decrease in the basic reproductive number when looking at dogs from ever positive sires and never positive mothers, could potentially indicate that the maternal role in vertical transmission is much higher, but there is still an impact from the sire. As the R_0_ does account for all modes of transmission, this R_0_ does not exclude potential horizontal (biting fighting) transmission within the population [41].

Though not significant, the sire’s qPCR status the year of the pups’ birth is trending towards being a protective factor for the pup. This potentially indicates a biological mechanism to prevent an active infection infecting future offspring, as it might be important that the sire has a high anti-*Leishmania* antibody titer to have parasites in the semen, while parasitemia somehow does not lead to positive seminiferous fluid. We do not honestly understand this fully, but it is of interest. This effect was only seen with a positive dam and sire; the dam’s diagnostic status is a more influential indicator of the offspring’s lifetime status. We will also restate that there is still a potential for unassessed bite/fight horizontal transmission perhaps could be related to this result or portions of it.

Alongside venereal transmission stemming from the sire, further research into pups born to mothers diagnostically negative for *L. infantum* and the dams themselves is necessary to elucidate *L. infantum* vertical transmission. As there is a report of venereal transmission of *L. infantum*, the sire could potentially infect a dam who later will vertically pass the infection on to her pups [18]. Another future direction would be used large scale xenodiagnoses in dog breeding programs with available immunotherapy treatments and vaccination for both parents to lower the parasite burden circulation before and during pregnancy that might permit the use of seropositive dogs from distinct bloodlines to be used as breeders but not infecting the offsprings [20]. In addition, this study shows the importance of testing male and females’ breeders serologically against *Leishmania* before entering in the breeding programs. Uninfected parents will have low risks transmitting *Leishmania* to the future blood lines. The testing is also critical to draft sires between kennels and prior to importation of dogs for breeding from highly infectious areas around the globe [9].

To control canine *L. infantum* infections, a One Health approach is necessary. As it is known that culling of infected dogs does not decrease infections among humans or dogs on its own, other methods must be used [5–7]. These methods include canine insecticide prophylactic treatment, the use of insecticides and bed nets to protect people and effective environmental vector management (EVM), such as removing sandfly breeding sites including decaying organic matter and waste around dwellings [2,6,8,42]. This study demonstrates the need for control venereal transmission through selective breeding of sires and dams.

## Supporting information

S1 Data Data used to analyze the experiments. (XLSX)
